# CT-RPL: Cluster Tree Based Routing Protocol to Maximize the Lifetime of Internet of Things

**DOI:** 10.3390/s20205858

**Published:** 2020-10-16

**Authors:** Sennan Sankar, Somula Ramasubbareddy, Ashish Kr. Luhach, Anand Nayyar, Basit Qureshi

**Affiliations:** 1 Department of Computer Science and Engineering, Sona College of Technology, Salem 636005, India; sankar.cse@sonatech.ac.in; 2 Department of Information Technology, VNR Vignana Jyothi Institute of Engineering & Technology, Hyderabad 500090, India; svramasubbareddy1219@gmail.com; 3 Department of Electrical and Communication Engineering, The PNG University of Technology, Lae 411, Papua New Guinea; ashishluhach@acm.org; 4 Graduate School, Duy Tan University, Da Nang 550000, Vietnam; 5 Faculty of Information Technology, Duy Tan University, Da Nang 550000, Vietnam; 6 Robotics and Internet of Things Lab, Prince Sultan University, Riyadh 12435, Saudi Arabia; qureshi@psu.edu.sa

**Keywords:** clustering methods, energy consumption, energy efficiency, game theory, Internet of Things, routing protocol

## Abstract

Energy conservation is one of the most critical challenges in the Internet of Things (IoT). IoT devices are incredibly resource-constrained and possess miniature power sources, small memory, and limited processing ability. Clustering is a popular method to avoid duplicate data transfer from the participant node to the destination. The selection of the cluster head (CH) plays a crucial role in gathering and aggregating the data from the cluster members and forwarding the data to the sink node. The inefficient CH selection causes packet failures during the data transfer and early battery depletion nearer to the sink. This paper proposes a cluster tree-based routing protocol (CT-RPL) to increase the life span of the network and avoid the data traffic among the network nodes. The CT-RPL involves three processes, namely cluster formation, cluster head selection, and route establishment. The cluster is formed based on the Euclidean distance. The CH selection is accomplished using a game theoretic approach. Finally, the route is established using the metrics residual energy ratio (RER), queue utilization (QU), and expected transmission count (ETX). The simulation is carried out by using a COOJA simulator. The efficiency of a CT-RPL is compared with the Routing Protocol for Low Power and Lossy Networks (RPL) and energy-efficient heterogeneous ring clustering routing (E2HRC-RPL), which reduces the traffic load and decreases the packet loss ratio. Thus, the CT-RPL enhances the lifetime of the network by 30–40% and the packet delivery ratio by 5–10%.

## 1. Introduction

In today’s world, the Internet of Things (IoT) provides a platform for the researchers to extend communication paradigm to new and varied levels. Computing and sensor devices in the IoT are connected to the Internet, and provide services anytime, anyplace, and anywhere [1,2,3,4,5]. It consists of homogeneous and heterogeneous systems and devices including computers, mobiles, laptop, home appliances, consumer electronics, sensors, and actuators [6,7,8,9,10]. The IoT devices and systems have been used to sense the location of the objects, relay sensor and location information, establish secure communication channels, create on-the-go ad-hoc infrastructures, and integrate with Cloud for the remote control and processing of information [11,12]. In many recent works, the IoT has been used in building automation, smart cities, smart farming, smart home, smart-retail management, and smart grid systems, among many more [13,14].

The low power and lossy networks (LLN) is an IoT network that includes low power, limited processing capacity, and limited on-board memory and communication capabilities [15,16,17]. The routing protocol for low power and lossy networks (RPL) is a standardized protocol for LLNs. The RPL is a distance-vector routing protocol, which supports point-to-multipoint, point-to-point, and multipoint-to-point traffic patterns in LLN [18,19]. In RPL, the parent selection is performed on the basis of the objective function (OF). The OF function defines the method of choosing the finest parent in the destination-oriented directed acyclic graph (DODAG). The RPL rank shows the participant node, how far from the root of the DODAG [20,21]. In RPL, rank indicates the number of hops between participant node and DODAG root. Initially, the RPL protocol is introduced with a single routing metric to pick the parent node in the DODAG. Many research works have focused on expanding the lifespan of the network, reducing the data traffic across the network, and improving the quality of services, hence improving the route stability during the data transmission. The standard RPL protocol is based on the hop count, which is called an objective function-0 (OF-0). The expected transmission count is a routing metric, which is used to pick the efficient parent for the quality of services. Although various parameters, including expected lifetime [22], load balance, residual energy, and sigma routing metric [23], are introduced to prolong the lifespan of the network and quality of service, the single metric-based routing selection alone is not effective. Recent works have focused on the multiple routing metrics for selecting the parent to establish an efficient route. These works consider the routing metric residual energy and battery depletion index [24], queue utilization and expected transmission count, survivability of path, congestion, and interference so as to extend the network lifetime in different scenarios [25]. The average weighted method is not suitable for composite routing metrics with maximization and minimization properties of the network node and link metrics [26]. Researchers in [27] used the composite routing metric-based routing selection using fuzzy logic. Fuzzy logic is applied on the routing metric’s expected transmission count and energy consumption, link quality level, end-to-end delay, hop count, and residual energy for selecting the best parent in the parent list. However, the composite routing metrics are only suitable for specific applications that support multi-hop routing.

A joint energy supply and routing path selection algorithm for WSN was proposed by Tang [28] to conserve the energy among the network nodes. The path selection algorithm adjusts the parameters dynamically that satisfy the network condition in order to extend the lifetime of the network. Zhou et al. proposed a hybrid architecture of distributed remote radio heads and a centralized baseband unit to improve the quality of service and conserve the energy in the network. It is noted that implementing the complex interference scenarios is challenging and takes more time for decision-making [29]. Moreover, the sensor devices can generate redundant data that may not have any useful information, all of which is forwarded to the DODAG root. As a solution, the authors in [30,31,32] used clustering as a necessary process for particular IoT applications, such as thermal monitoring, radiation monitoring, and weather monitoring.

Clustering is an essential process for extending the lifespan of the network [33]. Such protocols resolve many issues like energy efficiency, scalability, reliability, and the network lifetime. The sensor nodes are located randomly in the IoT network, and the clusters form the following criteria. The cluster head (CH) is elected from each cluster, based on certain parameters, including residual energy, link quality, and queue size. The CH nodes forward the data packets to the sink [34]. The low-energy adaptive clustering hierarchy (LEACH) protocol is a standard clustering protocol in WSN [35]. A drawback in LEACH is the uneven distribution of clusters due to the random placements of nodes in the network; as a result, cluster members (CM’s) are unable to send the data to the sink due to the CH node failure by the hotspot problem [36]. Researchers have worked on cluster-based RPL protocol and game theory-based CH selection for increasing the lifespan of IoT networks.

This paper presents how the cluster-tree-based routing protocol (CT-RPL) is an improvement over the existing work in the cluster formation process, cluster head selection process, and route establishment process. The cluster is constructed based on the Euclidean distance of network nodes. The game theoretic approach is used to perform the CH selection. The route establishment process is based on the metric queue utilization (QU) and expected transmission count (ETX) to select the optimal CH parent for efficient data transfer and to extend the lifespan of the network.

The main objectives of this paper are as follows.

- An in-depth analysis of the literature concerning various clustering protocol proposed by various researchers to enhance the performance parameters for the IoT network in terms of energy efficiency, packet delivery ratio, and end-end delivery.
- In CT-RPL, the virtual clusters are created using Euclidean distance. Later on, a game theoretic approach is proposed for selecting the effective CH node among the clusters. As a consequence, the nodes maintain effective energy utilization and exhibit a gradual depletion of the energy in the network nodes.
- Additionally, the CT-RPL reduces the network traffic and avoids a bottleneck problem at the root. It is achieved by considering the routing metrics expected transmission count (ETX), residual energy ratio (RER), and queue utilization (QU) for data transmission.
- The simulation is conducted, and the performance of the CT-RPL is compared with RPL and energy-efficient heterogeneous ring clustering routing (E2HRC-RPL). The CT-RPL increases the network lifetime and decreases the end-to-end delay.

The rest of the paper is organized as follows: Section 2 discusses the related works. Section 3 presents the cluster tree-based RPL (CT-RPL) using a game theoretical approach. The results and discussions are addressed in Section 4. Finally, the conclusion and future works of the paper are mentioned in Section 5.

## 2. Related Works

This section discusses various cluster-based routing protocols that can be used by the Internet of Things to increase the lifespan of the network.

Zhang et al. [37] proposed an energy-efficient heterogeneous ring cluster routing (E2HRC) protocol in WSNs, where a ring clustering is performed with event-driven CH rotation principle-based cluster head selection. However, E2HRC-RPL initially takes more time to form the clusters in the network. Conti et al. [38] created a set of RPL, resulting in the extension of the packet delivery ratio by 25%, increasing the scalability and data communication by reducing the latency and packet loss in IoT networks. On the other hand, this creates node congestion for unbalanced clusters in the network. A content-centric RPL (CCR-RPL) was proposed by Jin et al. [39] that reduced the data traffic across the network, using a higher data aggregation ratio in the routing process. Even though their proposed protocol outperformed the standard RPL in terms of latency and packet delivery ratio, it supported limited content being highly resource-constrained in the nodes.

Xu et al. [40] proposed an energy-efficient region source routing (ER-SR) protocol for WSN. The protocol distributed the energy utilization among the nodes to increase the lifespan while overcoming the limitations of resource constrain. ER-SR offered a substantial performance with respect to the energy efficiency, lifespan, and latency as compared to other RPL in WSN, but it required extra time for picking up a suitable parent for selecting CH in the cluster. Zhao et al. [41] proposed an energy-efficient region-based RPL (ER-RPL) for LLN. The protocol improved the latency and energy consumption but faced node congestion due to uneven region formation. A hierarchical-distributed management clustering (HDMC) protocol for WSN was designed by Shahraki [42] that surpassed the performance of LEACH and hierarchical-distributed management clustering (HEED) in terms of latency and lifetime. The model had limited applications, unlike the previously mentioned protocols. An adaptive distributed game theory-based congestion control in RPL for WSN was proposed by Ramesh and Priya [43] to solve the congestion issue of RPL. The protocol supported both congestion detection and moderation during the route establishment, increasing the network lifespan and decreasing the latency. However, the threshold value was not well-defined for the protocol. Lin and Wang [44] proposed an energy-efficient clustering protocol using dual cluster head and game theory (ECGD) in WSN. The protocol’s effectiveness was compared to LEACH-ERE, threshold-sensitive energy-efficient sensor network (TEEN), and power-efficient gathering in sensor information systems (PEGASIS). The ECGD protocol achieved superior performance through the network’s energy and lifespan of the network. Sohail et al. [45] proposed a game theoretic approach for power management (GTSPM) in the IoT. The selection of CH played an important part in the IoT network. It considered the game theoretical approach for selecting the cluster’s appropriate CH nodes. The effectiveness of GTSPM was compared with the LEACH and game theory-based energy-efficient clustering (GEEC) routing protocol. However, it provided less reliability compared to other protocols. Verma et al. [46] proposed two novel algorithms, namely the genetic algorithm-based optimized clustering (GAOC) protocol and data sink-based GAOC (MS-GAOC), for electing the CH node in WSN. It addressed the hotspot problem in the network. The GAOC protocol was compared with the dynamic cluster head selection based on the genetic algorithm (DCH-GA), genetic algorithm-based distance-aware LEACH (GADA-LEACH), and threshold-sensitive energy-efficient delay-aware routing protocol (TEDRP). Additionally, the MS-GAOC protocol was compared with the multiple data sink-based dynamic clustering of heterogeneous wireless sensor networks, based on a GA (MS-DCHGA), multiple data sink-based threshold-sensitive energy-efficient delay-aware routing protocol (MS-TEDRP), and multiple data sink-based genetic algorithm-based distance-aware routing (MS-GADA) protocol. However, its CH selection was inefficient due to the distributed approach of CH selection and haul transmission. Verma et al. [47] proposed two protocols, namely the multiple data sink-based energy-efficient cluster routing (MEEC) protocol and improved dual hop routing (IDHR) protocol, for electing the optimal CH selection in WSN. It concentrated mainly on the network longevity and network hotspot problem. The efficacy of the IDHR protocol was compared with the threshold-sensitive energy-efficient delay-aware routing protocol (TEDRP), stable energy-efficient clustering protocol (SEECP), and distance-based residual energy-efficient stable election protocol (DRESEP). The performance of the MEEC was also compared with the IDHR. Thus, it prolonged the network lifetime. However, the energy depletion increased due to the systematic rotation of the relay nodes and increased the consequently depleted energy. A novel routing architecture designed for harsh environment monitoring was proposed by Verma [48]. The objective of this work was to prolong the lifespan by addressing the hotspot problem in the network. The intra-cluster routing was improved by adopting node density factor in CH selection. Thus, it effectively extended the network as compared to the stable and energy-efficient clustering protocol (SEECP). However, other radio interferences were not considered in this work. Quality of Service (QoS) provisioning-based routing protocols using multiple data sinks in the IoT were proposed by Verma [49]. The cluster head (CH) selection was optimized by adopting the metrics, distance, node density, remaining energy, and energy threshold factors. The simulation results confirmed that the proposed protocol outperformed the state of the standard protocols. However, it was not suitable for large area networks. Verma et al. [50] also investigated various hierarchical routing protocols in WSN. They discussed the versatile routing protocols and their advantages and limitations. A new neuro fuzzy-based cluster formation protocol (FBCFP) for WSN-based IoT was proposed by Thangaramya [51]. The effectiveness of FBCFP was compared with LEACH and HEED. It demonstrated better performance by means of energy utilization, end-to-end delay, and lifespan of the network. Munisamy et al. [52] proposed virtual force-based intelligent clustering for energy-efficient routing (VFICEER) in WSN. The advantage of VFICEER increased the network lifetime and packet delivery ratio. Moreover, it reduced the delay and energy consumption. However, the energy consumption was high due to the ad-hoc nature of this network. Turgut [53] proposed a distributed clustering with decreased uncovered nodes (DiCDU) to resolve the uncovered node problem after the election of cluster heads. Thus, it extended the lifetime of the network. Dao et al. [54] proposed a new approach of aggregating data in CHs for improving the classification of support vector machines (SVM) with a flower pollination algorithm (IFPA) to achieve classification accuracy. The IFPA-SVM algorithm forwarded a number of data, which was high when compared to the SVM and hidden Markov model (HMM). However, the CH node in IFPA-SVM consumed more energy than the other algorithms. In the related works, various cluster-based RPL protocols, clustering protocols, and CH selection were discussed using a game theoretic approach.

The following are the limitations of the cluster-based RPL protocol: (i) it takes more time to form the clusters in the network, (ii) it results in network congestion and unbalanced energy among the nodes in the clusters, and (iii) it takes time to select the cluster parent in the network. It is further observed that the clustering protocol is suitable for specific applications, such as temperature monitoring and light event detection. Although researchers have proposed various techniques such as probability basis, fuzzy logic, and residual energy to optimize the energy utilization among the nodes in the cluster, nonetheless, there is still a gap in the literature that focuses on prolonging the network’s lifespan while balancing the energy utilization among the cluster nodes. Keeping all these existing works in consideration, the objective of this paper is to prolong the lifespan of the network. This paper proposes a cluster-tree-based routing protocol (CT-RPL) that performs cluster formation process, cluster head selection, and the route establishment process in order to increase the lifespan of the network.

## 3. Cluster Tree-Based Routing Protocol

The paper proposes a cluster tree-based routing protocol to enhance the lifespan of the network. CT-RPL involves three processes: (i) cluster formation, (ii) CH selection, and (iii) route establishment. The virtual clusters are created according to the Euclidean distance of the network nodes. The CH selection is carried out by using a game theoretic approach. Finally, the route establishment process uses the routing metrics, namely queue utilization and the expected transmission count, to pick the optimum CH parent for effective data transfer.

### 3.1. System Model

#### 3.1.1. Network Model

**Figure 1:**
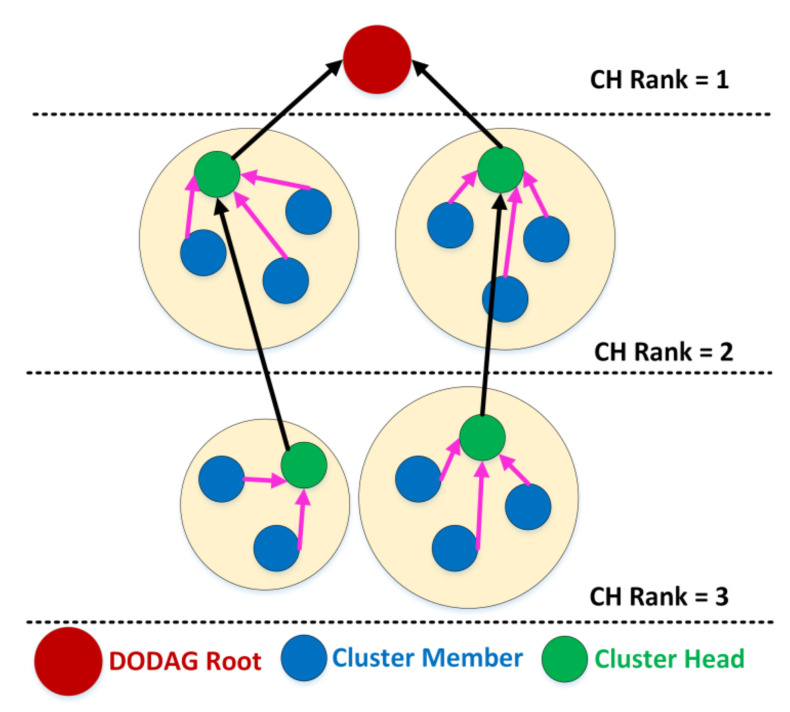
Cluster tree-based routing protocol (CT-RPL) network model. DODAG: destination-oriented directed acyclic graph.

The network area comprises of “N” nodes, which are placed randomly. The DODAG root is deployed on the top of the network. Initially, all the nodes have the same energy. Figure 1 shows the CT-RPL network model.

The following assumptions were made for CT-RPL.

- All the nodes are same types of nodes with equal initial energy.
- The nodes are stationary and located randomly in the network.
- The CT-RPL follows the multi-hop routing from the CH node to the DODAG root.
- All the cluster members in the cluster communicate with the DODAG root through the CH node.

#### 3.1.2. Energy Model

**Figure 2:**
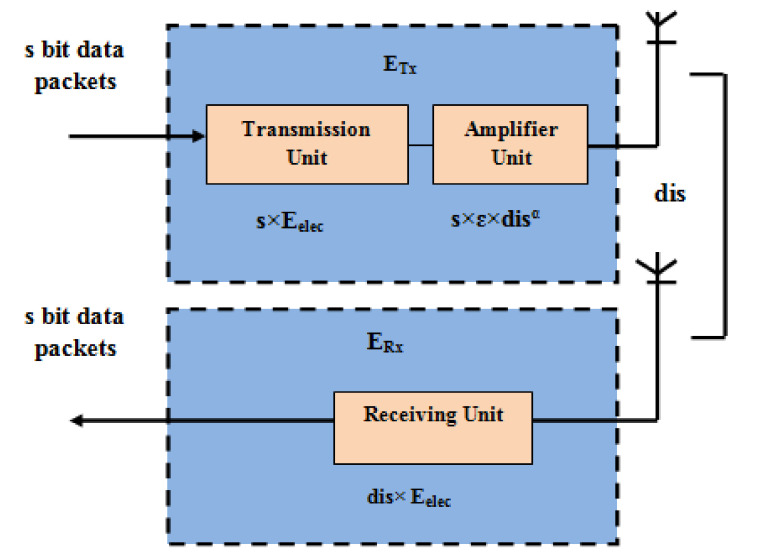
CT-RPL energy model.

The energy consumption model adopts the free space channel with $dis^{2}$ energy consumption and the multipath channel with $dis^{4}$ energy consumption, depending on the distance between the transmitter and the receiver. Figure 2 depicts the CT-RPL energy model.

The amount of energy required for transmitting “*s*” bits of data from node “*x*” to node “*y*” with respect to the distance *dis*(*x*,*y*) and its calculation is given in Equation (1) [55].The total transmission energy $E_{TX}$ calculation is given in Equation (1).

(1) $$E_{TX}\left(s,dis\right)=s\times E_{elec}+s\epsilon \times dis{\left(a,b\right)}^{\alpha }\;=\;\left\{\begin{array}{c} s\times E_{elec}+s\times {ɛ}_{fs}\times dis{\left(a,b\right)}^{2}\;\;\;where\;dis\left(a,b\right)<dis_{0}\\ s\times E_{elec}+s\times {ɛ}_{mp}\times dis{\left(a,b\right)}^{4}\;\;where\;dis\left(a,b\right)\geq dis_{0} \end{array}\right.$$

where $\varepsilon_{fs}$ energy dissipated in the amplifier while *dis* ≤ $dis_{0},$ and ${ɛ}_{mp}$ is the energy dissipated in the amplifier while *dis* ≥ $dis_{0}$, $E_{elec}$ is the consumed energy in the transmission unit, ε is a constant, and *s* is number of data bits.

The distance between the transmitter and receiver threshold ${dis}_{0}$ is given thus:

(2) $$dis_{0}=\sqrt{\frac{{ɛ}_{fs}}{{ɛ}_{mp}}}$$

The amount of energy needed for receiving s bits $E_{RX}$ of data packets from the participant RPL router is given thus:

(3) $$E_{RX}\left(dis\right)=dis\times E_{elec}$$

### 3.2. Cluster Formation

Each node in the network calculates the distance using Euclidean distance [56]. The distance “*dis*” between two nodes is calculated by

(4) $$dis=\sqrt{{{(x}_{j}{-x}_{i})}^{2}{+(y}_{j}{-y}_{i}{)}^{2}},$$

where ($x_{i},{\;y}_{i}$) and ($x_{j},\;y_{j}$) are coordinates of the network nodes “*i*” and “*j*”.

The distance between one node and all other nodes are calculated, and its matrix representation is given thus:

(5) $$dis\;=\;\left[\begin{array}{ccc} 0 & {dis}_{1,2}\ldots & {dis}_{1,n}\\ \vdots & 0\;\ldots & \vdots \\ {dis}_{n,1} & {dis}_{n,2\ldots } & 0 \end{array}\right]$$

The centroids are computed in the network nodes, and their calculation is given thus:

(6) $$centroid(\left(x_{i}{-y}_{i}),\;(x_{j}{-y}_{j}\right))\;=\;(\frac{x_{i}{+x}_{j}}{2},\frac{y_{i}{+y}_{j}}{2})$$

Initially, the coordinates of the first node are known to be the centroid point. First, the CT-RPL measures the distance “*dis*” between two nodes and, also, finds out the centroid of both nodes. Later, it picks the next node’s coordinates and the recently computed centroid point for calculating the new centroid point. If the centroid values are within the distance “*dis*” value, the node is added in the same cluster; otherwise, it will be added in the distinct cluster.

The number of clusters is computed, and its calculation is given in Equation (7).

(7) $$C_{i}\;=\;\left\{\begin{array}{c} {Same\;cluster,\;centroid(x}_{i}{,\;y}_{i})\;\pm \;dis\\ \mathrm{Distinct}\;cluster,\;otherwise \end{array}\right.$$

The total number of clusters “*C*” is calculated from Equation (8).

(8) $$C\;=\;\overset{m}{\underset{k=1}{\sum }}count(centroid\left(x_{i}{,\;y}_{i}\right))$$

where *m* indicates the number of nodes in each cluster.

### 3.3. Cluster Head Selection

In Game theory, a non cooperative game contains a set of players N, set of actions U to take a decision for each player, and the payoff p is a reward or penalty for each player [57]. Nash equilibrium (NE) is a decision state, when the player reaches the outcome. Each node in the cluster is treated as a set of players in the game. It has a set of actions U such as residual energy, sense, receive, aggregate, transmitting the data from one to other node, and number of times the node is selected as a CH in a cluster. Each player is not only affected by its own actions but, also, gets affected by other player’s action. At the beginning of the game, the sensor nodes have the same energy.

The energy consumption of the node is computed using the radio energy model. The amount of energy required to transfer “*s*” bits of data packets with respect to the distance “*dis*” is calculated, and it is given in Equation (9).

(9) $$ET_{x}\left(s,\;dis\right)=s\times E_{elec}+s\times \epsilon f_{s}\times dis^{2}$$

where *s*, *dis*, $E_{elec,}$ and $\epsilon f_{s}$ indicate the number of bits, electronic energy in the network node, distance between two nodes, and free space model, respectively.

An amount of energy is required to collect the data at each node in the cluster, and it is given in Equation (10).

(10) $$ER_{x}\left(r\right)=s\times E_{elec}$$

where $E_{elec}$ is the electronic energy in the RPL router, and *r* is a number of bits.

The amount of energy is depleted $E_{\mathrm{dep}}$ at each node in the cluster, and it is given by:

(11) $$E_{dep}{=E}_{sense}{\;+\;E}_{receive}{\;+\;E}_{aggregation}{\;+\;E}_{transmit}$$

where $E_{sense}$, $E_{receive}$, $E_{aggregation}$, and $E_{transmit}$ indicate the sensing energy, receiving energy, aggregation energy, and transmission energy, respectively.

The number of times a *CM* node is selected as the *CH* node in a cluster ${\;N}_{{CM}_{k,l}}^{CH}$ is given in Equation (12).

(12) $${\;N}_{{CM}_{k,l}}^{CH}=\frac{{\mathrm{Number}\;\mathrm{of}\;\mathrm{times}\;CM}_{k,l}\;\mathrm{node}\;\mathrm{act}\;\mathrm{as}\;CH}{\mathrm{Total}\;\mathrm{number}\;\mathrm{of}\;\mathrm{round}}$$

where *k* and *l* indicate kth cluster and lth node in a cluster, and “*N*” indicates the number of times.

The reward value is provided for each *CM* node on the basis of probability values, and its values are represented in Table 1.

Each cluster contains “*m*” number of nodes. Each node calculates the payoff value $p\left({CM}_{k,l}\right)$. The ${p(CM}_{i,j})$ value is a reward or penalty of a particular node action in the game, and its calculation is given in Equation(13).

(13) $$p\left({CM}_{k,l}\right){\;=\;R}_{k,l}{\times RER(CM}_{k,l}{)-P}_{k,l}{\times E}_{dep}{(CM}_{k,l})\;+\;R(N_{CM_{k,l}}^{CH})$$

where $R_{k,l}$ is a reward value of the lth node in a cluster *k*, $P_{k,l}$ is a penalty value of lth node in the cluster k, ${RER(CM}_{k,l})$ indicates the residual energy of the lth node in the cluster k, $E_{dep}\left(CM_{k,l}\right)$ indicates the energy depletion ratio (EDR) in the lth node in the cluster *k*, and ${R(N}_{{CM}_{k,l}}^{CH})$ indicates the reward value for the *CM* node acting as the *CH* node in the lth node in the cluster “*k*”.

The reward and penalty values range between 0 and 100. The residual energy ratio (*RER*) and energy depletion ratio (EDR) range between 0% and 100%. The initial energy of the node value represents 255, and the dead node value is to be 0. The residual energy is calculated from the proportion between the consumed energy and initial energy. The reward and penalty values are calculated from the RER and EDR that is given in Table 2.

The Nash equilibrium is a decision state in game theory. In each cluster, the node with the maximum payoff “*p*” value acts as a *CH* node in that particular round. The *CH* node calculation in that particular round is computed in Equation (14).

(14) $${CH}_{k,l}=\underset{k=1\;\mathrm{to}\;C}{max}\underset{l=1\;\mathrm{to}\;m}{max}{p(CM}_{k,l})$$

where ${p(CM}_{k,l})$ indicates the payoff value of the lth *CM* in the cluster *k*, m indicates the total number of nodes in the cluster, and *C* indicates the total number of clusters in the network.

### 3.4. Route Establishment

In CT-RPL, the route is established based on the standard RPL protocol. It uses queue utilization (*QU*), expected transmission count (*ETX*), residual energy (*RER*), and the cluster with the highest node count to pick the optimum *CH* for effective data transfer. Thus, CT-RPL deals with the hotspot problem effectively to a certain extent, while adding metrics to balance the load among the *CH* nodes in the network. In general, the DODAG root sends the DIOC message frequently to nearby CH nodes. The participant *CH* node receives the parent information, such as the *ETX* and *QU*, through the DIOC control message. The participant *CH* sends the DAOC to its parent. The DODAG root sends the DODAG Advertisement Object Cluster-Acknowledgement (DAOC-ACK) message to the participant *CH* node. Once the route is created from the participant to the root node, the *CH* receives its *CM* data and forwards it to their respective parent *CH*.

While establishing the route, routing metrics, objective function, and *CH* ranking calculations are crucial to assess the performance of the selection. These parameters are defined in the following subsection.

#### 3.4.1. Routing Metrics

The queue utilization (*QU*) and expected transmission count (*ETX*) are two metrics for routing performance. *QU* is a proportion between the number of packets occupied in the *l*th node’s queue ${QU}_{occupied}\left(l\right)$ and the total number of available packets in the *i*^th^ node’s queue ${QU}_{total}$ [58]. Its calculation is given in Equation (15).

(15) $$QU(l)=\frac{{QU}_{occupied}\left(i\right)}{QU_{total}\left(i\right)}$$

*ETX* indicates the link quality between the nodes. It is the proportion between the number of data transmissions and the retransmissions that are required to send the data successfully from node “*l*” to node “*p*”. It is given by:

(16) $$ETX(l,p)=\frac{1}{F_{d}{\;\times \;R}_{d}}$$

where $F_{d}$ indicates the forward data delivery, and $R_{d}$ indicates the reverse data delivery or acknowledgement from the receiver.

*RER* denotes the present energy available in the network nodes. The *RER* calculation is given in Equation (17).

(17) $$RER\left(CH_{k,l}\right)=\frac{E_{present}}{E_{initial}}\times 100$$

where *RER*($CH_{k,l}$) indicates the *CH* node residual energy in *l*th node of cluster *k*, and $E_{present}$ and $E_{initial}$ indicate the present energy and initial energy in the node, respectively.

The cluster with the highest node count (*CHNC*) is the ratio between total number of nodes present in the *i*th cluster and total number of nodes present in the network. The *CHNC* calculation is given in Equation (18).

(18) $$CNHC\left(CH_{k}\right)=\frac{T_{c}}{T_{N}}$$

where $T_{c}$ and $T_{N}$ indicate total number of nodes present in the *k*th cluster and the total number of nodes in the network, respectively.

#### 3.4.2. Objective Function

It helps to find the optimal route among the multiple routes in the DODAG. In CT-RPL, the objective function of node i ${OF}_{\left(QU,ETX\right)}\left(l\right)$ is calculated from the routing metrics queue utilization and expected transmission count. CT-RPL provides better results when the $w_{1}$, $w_{2}$, $w_{3},$ and $w_{4}$ weight values are 0.25, 0.25, 0.25, and 0.25, respectively. The *OF* calculation is given in Equation (19).

(19) $$OF_{\left(QU,ETX\right)}\left(l\right)=w_{1}\times QU\left(l\right)+w_{2}\times ETX\left(l\right)+w_{3}\times RER\left(l\right)+w_{4}\times CTHC\left(l\right)$$

#### 3.4.3. CH Rank Calculation

The *CH* rank exhibits the number of hops between the DODAG root and participant *CH*. The rank of *CH*, ${CH}_{r}$ of node *X*, is computed from the rank increase value, $r_{i}$ and the *CH* parent rank $r_{p}$ of *X*. $r_{i}$ is the summation of step value $s_{v}$ and minimum hop rank increase $r_{min}$. The value of $r_{i}$ is fixed, which is 256 [59]. $s_{v}$ is calculated from the objective function $OF_{\left(QU,ETX\right)}$.

(20) $${CH}_{r\;}\left(X\right){=r}_{p\;}\left(X\right){+r}_{i}$$

(21) $$r_{i}{=\;s}_{v\;}{+r}_{min\;}$$

In CT-RPL, the cluster formation computational complexity is O(CDK), where C, D, and K indicate the number of clusters, dimensions of each point to be clustered, and the number of nodes to cluster. The computational complexity of the *CH* selection and multi-hop route establishment are O(n^3) and O(n). The overall computational complexity is O(n^3). The overall working mechanism of CT-RPL is given in Algorithm 1.
**Algorithm 1** Cluster Tree-Based Routing AlgorithmInput: Coordinates of Node “*N*”Output: Number of *CH* nodeCluster Formation:1: Calculate the distance between the nodes.$dist=\sqrt{{{(x}_{j}{-x}_{i})}^{2}{+(y}_{j}{-y}_{i}{)}^{2}}$2: Compute the distance matrix for all the nodes.$dist=\left[\begin{array}{ccc} 0 & t_{1,2}\ldots & t_{1,n}\\ \vdots & 0\;\ldots & \vdots \\ t_{n,1} & t_{n,2\ldots } & 0 \end{array}\right]$3: Calculate the centroid of the network nodes$centroid(\left(x_{i}{-y}_{i}),\;(x_{j}{-y}_{j}\right))=\;(\frac{x_{i}{+x}_{j}}{2},\frac{y_{i}{+y}_{j}}{2})$4: Generate the cluster in the network from Equation (7). 5: Calculate the total number of clusters *C*$C=\overset{x}{\underset{i=1}{\sum }}count(centroid\left(x_{i}{,y}_{i}\right))$6: Obtain a set of “*C*” clusters.*CH* Selection:7: Compute $E_{RER}$ and $E_{dep}$ of nodes in the cluster.8: Compute the payoff “*p*” value for each *CM*$p\left({CM}_{ij}\right)=R_{ij}\times RER\left({CM}_{j}\right)\;-{\;P}_{ij}\times E_{dep}\left({CM}_{j}\right)+R\left(N_{{CM}_{i,j}}^{CH}\right)$.9: Compute the Nash equilibrium for deciding the *CH* node in the cluster ${CH}_{i}=\underset{i=1\;tok}{max}p({CM}_{i})$10: Obtain the optimum *CH* node.Route Establishment:11: Compute the DIOC-ETX, DIOC-QU, DIOC-RER, and DIOC-CHNC from the parent *CH* node.12: Calculate the quality of the *CH* parent node using the objective function${OF}_{\left(QU,ETX\right)}\left(i\right)=w_{1}\times QU\left(i\right)+w_{2}\times ETX\left(i\right)+w_{1}\times RER\left(i\right)+w_{2}\times CHNC\left(i\right)$13: Compute the rank for selecting best *CH* parent node.${CH}_{r\;}\left(X\right)=r_{p\;}\left(X\right)+r_{i\;}$$r_{i\;}={\;s}_{v\;}+r_{min\;}$14: Return the best *CH* parent node.


**Figure 3:**
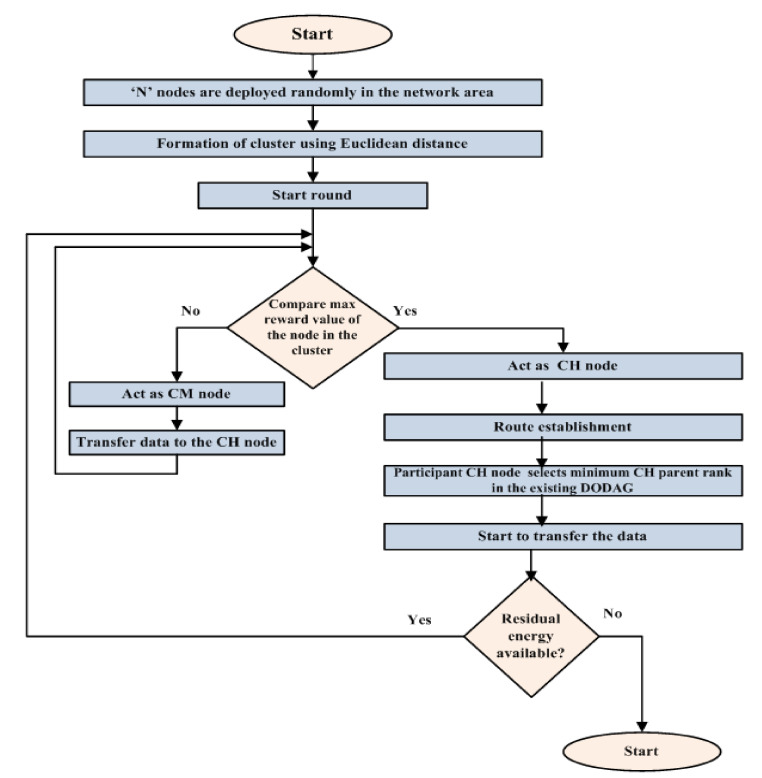
Working principle of CT-RPL.

The working mechanism of the cluster tree-based routing protocol (CT-RPL) is given in Figure 3:

After the deployment of nodes and the formation of clusters, the maximum reward values are compared, and the minimum *CH* parent rank is chosen after route establishment, based on which data transfer occurs, until the residual energy diminishes.

## 4. Results and Discussion

The purpose of the simulation is to examine the effectiveness of CT-RPL. Contiki COOJA simulator version 3.0 was utilized for the simulation. In this simulation, the number of RPL routers is taken to be 100 and DODAG root node to be 1. The simulation’s duration is one hour. The simulation network area is 200 × 200 m^2^. The value of the RPL parameter is 256, which is the default value. Table 3 displays the simulation settings for the research.

### 4.1. Number of Parent Node Changes

**Figure 4:**
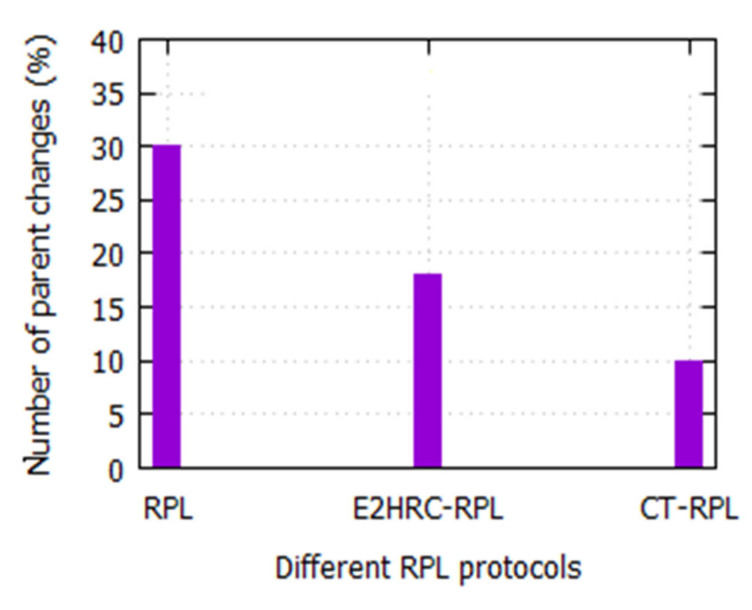
Number of parent node changes vs. various routing protocols (RPL). E2HRC-RPL: energy-efficient heterogeneous ring clustering routing protocol and CT-RPL: cluster tree-based routing protocol.

It indicates the number of times RPL routers change its parent during the simulation. Additionally, the parent changes denote the route stability among the network nodes. Figure 4 exhibits the number of parent changes for various RPL protocols. It is observed that the parent changes of RPL, E2HRC-RPL, and CT-RPL are 30%, 18%, and 10%, respectively. It is also noted that the parent change in the CT-RPL protocol is low when compared to RPL and E2HRC-RPL. It is due to the efficient rotation of cluster heads between cluster nodes and prevents redundant data transmission in the network nodes.

### 4.2. Packet Loss Ratio

**Figure 5:**
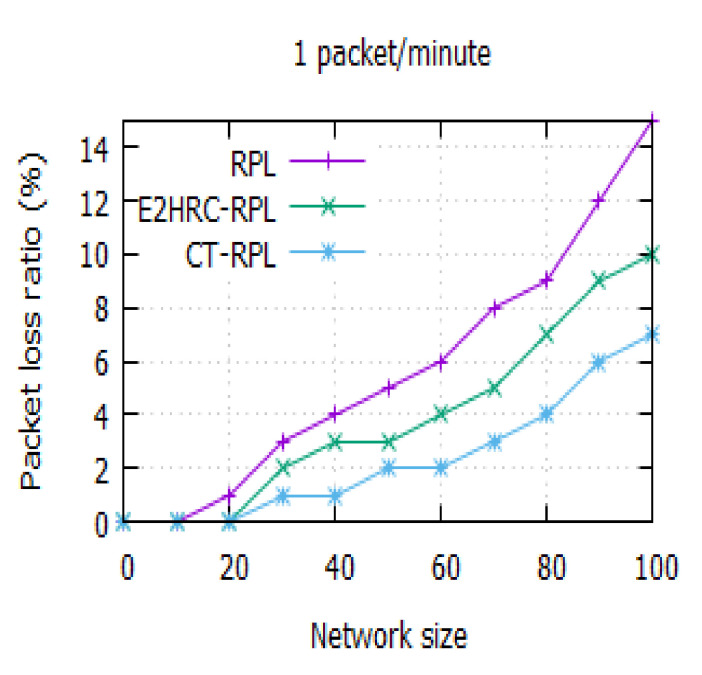
Packet loss ratio vs. network size for a network size of 100; the packet loss ratio of RPL, E2HRC-RPL, and CT-RPL are 15%, 10%, and 7%, respectively.

The packet loss ratio shows the proportion of the number of packets received out of the total number of packets sent. It also indicates the reliability of the data from the participant to the DODAG root. Figure 5 depicts the packet loss ratio for the different network nodes. The packet loss ratio of RPL, E2HRC-RPL, and CT-RPL are 15%, 10%, and 7%, respectively, for the network size of 100. It reveals that the packet loss ratio of CT-RPL increases with the increase in the size of the network node. It is owing to the consideration of the metrics *QU*, *ETX*, *RER*, and *CHNC* during the route establishment. It also results in reducing the path breakages in the data transmission.

The packet loss ratio for different network sizes is represented in Table 4. In this table, a comparison is made with the different routing protocols, namely RPL, E2HRC-RPL, and CT-RPL. Table 4 indicates that the packet loss ratio is low in CT-RPL as compared to RPL and E2HRC-RPL. The proposed CT-RPL reduces the packet loss ratio by half when the number of network size is 100.

### 4.3. End-to-End Delay

**Figure 6:**
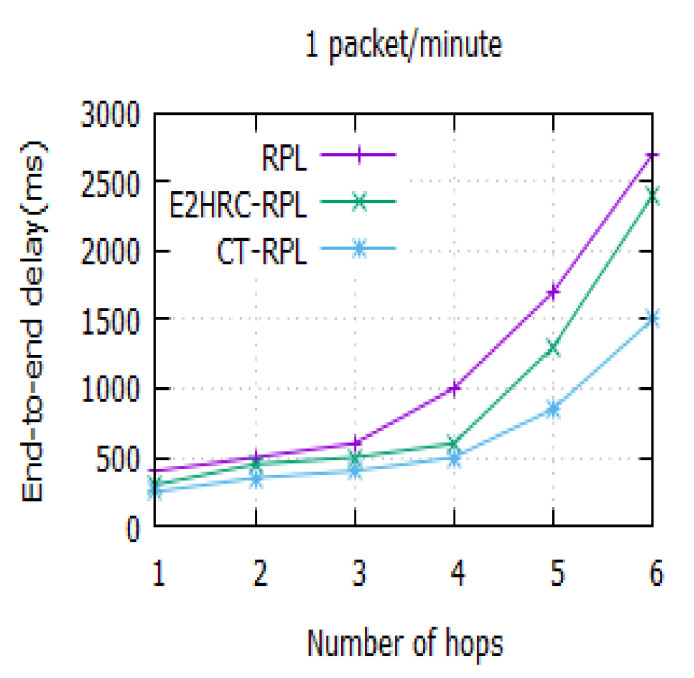
End-to-End delay vs. the number of hops for a hop count of 6; the delays of RPL, E2HRC-RPL, and CT-RPL are 2700 ms, 2400 ms, and 1500 ms, respectively.

The end-to-end delay indicates the amount of time taken for transmitting the sensor data between the RPL router and DODAG root. Figure 6 illustrates the end-to-end delay for a varying number of hops. It is observed that the delays of RPL, E2HRC-RPL, and CT-RPL are 2700 ms, 2400 ms, and 1500 ms, respectively, for the hop count of six. It is also noted that the node’s end-to-end delay increases for the varying number of hops. It is owing to the formation of clusters in the network nodes, and it applies the game theory for CH selection. Additionally, it considers the metrics *QU*, *ETX*, *RER*, and *CHNC* for the route establishment.

Table 5 indicates the end-to-end delay in RPL, E2HRC-RPL, and CT-RPL for a varying number of hops. The result confirms that the cluster-based routing protocols take less time comparatively when the number of rounds in the network is increased.

### 4.4. Energy Consumption

**Figure 7:**
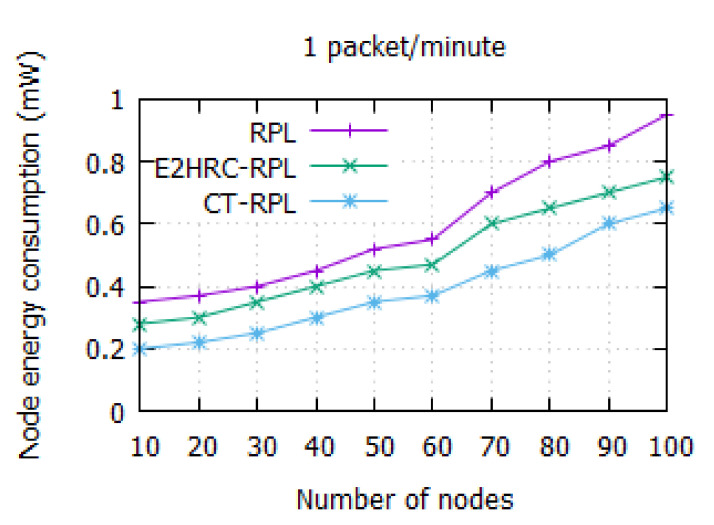
Node energy consumption vs. the number of nodes for a network size of 100; energy consumption of RPL, E2HRC-RPL, and CT-RPL is 0.95, 0.75, and 0.65 mW, respectively.

It indicates the energy consumption of network nodes. Figure 7 shows the node energy consumption relative to the network nodes. The energy consumption of RPL, E2HRC-RPL, and CT-RPL is observed to be 0.95 mW, 0.75 mW, and 0.65 mW, respectively, for the network size of 100. It is noted that the amount of energy dissipation increases as the number node increases. In CT-RPL, the network nodes consume less energy as compared to RPL and E2HRC-RPL. It is due to the efficient cluster head selection using a game theoretic approach. Additionally, the CT-RPL considers the metrics, namely *QU*, *ETX*, *RER*, and *CHNC*, to choose the optimum *CH* nodes in the parent *CH* list. It balances the network traffic among the *CH* nodes. Thus, it prolongs the network lifetime, and it solves the hotspot problem to a certain extent.

Table 6 indicates the node energy consumption among the network nodes. The energy consumption in CT-RPL is less as compared to other popular routing protocols, namely RPL and E2HRC-RPL. It explicitly shows that cluster-based routing consumes less power than the multi-hop routing protocol.

## 5. Conclusions and Future Works

The selection of the cluster head (*CH*) plays a crucial role in IoT devices by gathering and aggregating data from the cluster members and forwarding the data to the sink node. Inefficient *CH* selection causes packet failures during the data transfer and early battery depletion nearer to the sink. In this paper, a cluster tree-based routing protocol (CT-RPL) was proposed for addressing this issue. The purpose was served by the simulation of three processes. First, the virtual cluster was created according to the Euclidean distance. Later, the game theoretic approach was used to pick the *CH* node. Finally, the metrics QU and *ETX* were used to pick the optimum *CH* parent for effective data transfer. CT-RPL’s efficiency was compared to RPL and E2HRC-RPL. The CT-RPL showed an enhancement of the lifetime of the network by 30–40% and the packet delivery ratio by 5–10%.

In future research, the sky mote will be deployed in real time, and the performance of the CT-RPL protocol will be carried out in different scenarios.

## Figures and Tables

**Table 1:** Reward value for the cluster member (*CM*) node acting as the cluster head (*CH*) node.

$\mathrm{Probability}\;\mathrm{Value}\;\mathrm{of}\;\mathrm{CM}\;\mathrm{Node}\;\mathrm{Acting}\;\mathrm{as}\;\mathrm{CH}\;\mathrm{Node}\;N_{CM_{k,l}}^{CH}$	$\mathrm{Reward}\;R\left(N_{CM_{k,l}}^{CH}\right)$
0	100
0.2	80
0.4	60
0.6	40
0.8	20
1	0

**Table 2:** Reward and penalty value for each node.

Ratio (*RER*) (%)	$\mathrm{Reward}\;(R_{k,l})$	Energy Depletion Ratio (EDR) (%)	$\mathrm{Penalty}\;(P_{k,l})$
0–10	0	0–10	100
11–20	20	11–20	80
21–40	40	21–40	60
41–60	60	41–60	40
61–80	80	61–80	20
81–100	100	81–100	0

**Table 3:** Simulation parameters.

Parameter	Value
OS	Contiki 3.0
Routing Protocol	RPL
Node Type	Sky mote
Simulation Duration	1 h
MAC Layer	Contiki MAC/6LowPAN
Number of Nodes	100 RPL Router + DODAG root
Transmission Range	200 m × 200 m
Data Packet Timer	60 sec
Full Battery	3000 mJ
RPL Parameter	Minimum Hop Rank Increase = 256
Queue Size	8 packets
Data rate	1 pkt per min
Aggregation ratio ρ	0.8

**Table 4:** Packet loss ratio for different nodes. RPL: routing protocols, E2HRC-RPL: energy-efficient heterogeneous ring clustering routing protocol, and CT-RPL: cluster tree-based routing protocol.

Number of Nodes	Packet Loss Ratio (%)		
	RPL	E2HRC-RPL	CT-RPL
10	0	0	0
20	1	0	0
30	3	2	1
40	4	3	1
50	5	3	2
60	6	4	2
70	8	5	3
80	9	7	4
90	12	9	6
100	15	10	7

**Table 5:** End-to-End delay with respect to the number of hops.

Number of Hops	End-to-End Delay (ms)		
	RPL	E2HRC-RPL	CT-RPL
1	400	300	250
2	500	450	350
3	600	500	400
4	1000	600	500
5	1700	1300	850
6	2700	2400	1500

**Table 6:** Node energy consumption with respect to different nodes.

Number of Nodes	Node Energy Consumption (mW)		
	RPL	E2HRC-RPL	CT-RPL
10	0.35	0.28	0.20
20	0.37	0.30	0.22
30	0.40	0.35	0.25
40	0.45	0.40	0.30
50	0.52	0.45	0.35
60	0.55	0.47	0.37
70	0.7	0.6	0.45
80	0.8	0.65	0.5
90	0.85	0.7	0.6
100	0.95	0.75	0.65

## References

[1] Young G.O., Peters J. (1964). Synthetic Structure of Industrial Plastics in Plastics.

[2] De Matos E., Tiburski R.T., Moratelli C.R., Johann S., Amaral L.A.F., Ramachandran G., Hessel F. (2020). Context information sharing for the Internet of Things: A survey. Comput. Netw..

[3] Sennan S., Balasubramaniyam S., Luhach A.K., Ramasubbareddy S., Chilamkurti N., Nam Y. (2019). Energy and Delay Aware Data Aggregation in Routing Protocol for Internet of Things. Sensors.

[4] Luhach A.K., Luhach A.K. (2016). Analysis of lightweight cryptographic solutions for internet of thing. Indian J. Sci. Technol..

[5] Bouzebiba H., Mohamed L. (2020). FreeBW-RPL: A New RPL Protocol Objective Function for Internet of Multimedia Things. Wirel. Pers. Commun..

[6] Ben-Daya M., Elkafi H., Zied B. (2019). Internet of things and supply chain management: A literature review. Int. J. Prod. Res..

[7] Srinivasan C.R., Rajesh B., Saikalyan P., Premsagar K., Yadav E.S. (2019). A review on the different types of Internet of Things (IoT). J. Adv. Res. Dyn. Control. Syst..

[8] Chen S., Xu H., Liu D., Hu B., Wang H. (2014). A vision of IoT: Applications, challenges, and opportunities with china perspective. IEEE Internet Things J..

[9] Bandyopadhyay D., Sen J. (2011). Internet of things: Applications and challenges in technology and standardization. Wirel. Pers. Commun..

[10] Li Y., Alqahtani A., Solaiman E., Perera C., Jayaraman P.P., Buyya R., Ranjan R. (2019). IoT-CANE: A unified knowledge management system for data-centric Internet of Things application systems. J. Parallel Distrib. Comput..

[11] Asghari P., Rahmani A.M., Javadi H.H.S. (2019). Internet of Things applications: A systematic review. Comput. Netw..

[12] Kotsiou V., Papadopoulos G.Z., Zorbas D., Chatzimisios P., Theoleyre F. (2019). Blacklisting-based channel hopping approaches in low-power and lossy networks. IEEE Commun. Mag..

[13] Ganesh D.R., Patil K.K., Suresh L. (2019). Q-frpml: Qos-centric fault-resilient routing protocol for mobile-wsn based low power lossy networks. Wirel. Pers. Commun..

[14] Sanshi S., Jaidhar C.D. (2019). Enhanced mobility aware routing protocol for low power and lossy networks. Wirel. Netw..

[15] Bouaziz M., Rachedi A., Belghith A. (2019). EKF-MRPL: Advanced mobility support routing protocol for internet of mobile things: Movement prediction approach. Future Gener. Comput. Syst..

[16] Bouaziz M., Rachedi A., Belghith A., Berbineau M., Al-Ahmadi S. (2019). EMA-RPL: Energy and mobility aware routing for the Internet of Mobile Things. Future Gener. Comput. Syst..

[17] Safara F., Souri A., Baker T., al Ridhawi I., Aloqaily M. (2020). PriNergy: A priority-based energy-efficient routing method for IoT systems. J. Supercomput..

[18] Homaei H.M., Salwana E., Shamshirband S. (2019). An Enhanced Distributed Data Aggregation Method in the Internet of Things. Sensors.

[19] Iova O., Theoleyre F., Noel T. (2015). Using multiparent routing in RPL to increase the stability and the lifetime of the network. Ad. Hoc. Netw..

[20] Sanmartin P., Rojas A.A., Fernandez L., Avila K., Jabba D., Valle S. (2018). Sigma routing metric for RPL protocol. Sensors.

[21] Hassan A., Alshomrani S., Altalhi A., Ahsan S. (2016). Improved routing metrics for energy constrained interconnected devices in low-power and lossy networks. J. Commun. Netw..

[22] Abreu C., Ricardo M., Mendes P.M. (2014). Energy-aware routing for biomedical wireless sensor networks. J. Netw. Comput. Appl..

[23] Sankar S., Srinivasan P. (2018). Fuzzy logic based energy aware routing protocol for Internet of Things. Int. J. Intell. Syst. Appl..

[24] Gaddour O., Koubâa A., Abid M. (2015). Quality-of-service aware routing for static and mobile IPv6-based low-power and lossy sensor networks using RPL. Ad. Hoc. Netw..

[25] Tang L., Cai J., Yan J., Zhou Z. (2018). Joint energy supply and routing path selection for rechargeable wireless sensor networks. Sensors.

[26] Zhou Z., Dong M., Ota K., Wang G., Yang L.T. (2015). Energy-efficient resource allocation for D2D communications underlaying cloud-RAN-based LTE-A networks. IEEE Internet Things J..

[27] Shanthi G., Sundarambal M. (2019). FSO–PSO based multihop clustering in WSN for efficient medical building management system. Clust. Comput..

[28] Rajpoot P., Dwivedi P. (2019). Multiple parameter based energy balanced and optimized clustering for WSN to enhance the Lifetime using MADM Approaches. Wirel. Pers. Commun..

[29] Gupta P., Sharma A.K. (2020). Clustering-based heterogeneous optimized-HEED protocols for WSNs. Soft Comput..

[30] Ranganathan A., Rangasamy B. (2019). Analysis of Energy-Efficient Clustering Algorithms for Wireless Sensor Network (WSN). J. Test. Eval..

[31] Wang Q., Lin D., Yang P., Zhang Z. (2019). An energy-efficient compressive sensing-based clustering routing protocol for WSNs. IEEE Sens. J..

[32] Ren Q., Yao G. (2020). An Energy-Efficient Cluster Head Selection Scheme for Energy-Harvesting Wireless Sensor Networks. Sensors.

[33] Fu C., Jiang Z., Wei W.E.I., Wei A. (2013). An energy balanced algorithm of LEACH protocol in WSN. Int. J. Comput. Sci. Issues.

[34] Maheswari D.U., Sudha S. (2019). Node Degree based Energy Efficient Two-level Clustering for Wireless Sensor Networks. Wirel. Pers. Commun..

[35] Lee J.G., Chim S., Park H.H. (2019). Energy-Efficient Cluster-Head Selection for Wireless Sensor Networks Using Sampling-Based Spider Monkey Optimization. Sensors.

[36] Murugadass G., Sivakumar P. (2020). A hybrid Elephant Herding Optimization and Cultural Algorithm for Energy-Balanced Cluster Head Selection Scheme to extend the lifetime in WSNs. Int. J. Commun. Syst..

[37] Zhang W., Li L., Han G., Zhang L. (2017). E2HRC: An energy-efficient heterogeneous ring clustering routing protocol for wireless sensor networks. IEEE Access.

[38] Conti M., Kaliyar P., Lal C. (2019). Reliable Group Communication Protocol for Internet of Things. arXiv.

[39] Jin Y., Gormus S., Kulkarni P., Sooriyabandara M. (2016). Content centric routing in IoT networks and its integration in RPL. Comput. Commun..

[40] Xu C., Xiong Z., Zhao G., Yu S. (2019). An Energy-Efficient Region Source Routing Protocol for Lifetime Maximization in WSN. IEEE Access.

[41] Zhao M., Ho I.W.H., Chong P.H.J. (2016). An energy-efficient region-based RPL routing protocol for low-power and lossy networks. IEEE Internet Things J..

[42] Shahraki A., Rafsanjani M.k., Saeid A.B. (2017). Hierarchical distributed management clustering protocol for wireless sensor networks. Telecommun. Syst..

[43] Ramesh T., Priya S.S. (2018). Adaptive Distributed Game Theory Based Congestion Moderation in RPL Networks. J. Netw. Commun. Emerg. Technol..

[44] Lin D., Wang Q. (2019). An energy-efficient clustering algorithm combined game theory and dual-cluster-head mechanism for WSNs. IEEE Access.

[45] Sohail M., Khan S., Ahmad R., Singh D., Lloret J. (2019). Game Theoretic Solution for Power Management in IoT-Based Wireless Sensor Networks. Sensors.

[46] Verma S., Sood N., Sharma A.K. (2019). Genetic Algorithm-based Optimized Cluster Head selection for single and multiple data sinks in Heterogeneous Wireless Sensor Network. Appl. Soft Comput..

[47] Verma S., Sood N., Sharma A.K. (2019). A novelistic approach for energy efficient routing using single and multiple data sinks in heterogeneous wireless sensor network. Peer Peer Netw. Appl..

[48] Verma S., Sood N., Sharma A.K. (2019). QoS provisioning-based routing protocols using multiple data sink in IoT-based WSN. Mod. Phys. Lett. A.

[49] Verma S., Sood N., Sharma A.K. (2018). Design of a novel routing architecture for harsh environment monitoring in heterogeneous WSN. IET Wirel. Sens. Syst..

[50] Verma S., Mehta R., Sharma D., Sharma K. (2013). Wireless sensor network and hierarchical routing protocols: A review. Int. J. Comput. Trends Technol..

[51] Thangaramya K., Kulothungan K., Logambigai R., Selvi M., Ganapathy S., Kannan A. (2019). Energy aware cluster and neuro-fuzzy based routing algorithm for wireless sensor networks in IoT. Comput. Netw..

[52] Munuswamy S., Saravanakumar J.M., Sannasi G., Harichandran K.N., Arputharaj K. (2018). Virtual force-based intelligent clustering for energy-efficient routing in mobile wireless sensor networks. Turk. J. Electr. Eng. Comput. Sci..

[53] Turgut I.A. (2020). DiCDU: Distributed clustering with decreased uncovered nodes for WSNs. IET Commun..

[54] Dao T.K., Nguyen T.T., Pan J.S., Qiao Y., Lai Q.A. (2020). Identification Failure Data for Cluster Heads Aggregation in WSN Based on Improving Classification of SVM. IEEE Access.

[55] Zhang W., Guangjie H., Yongxin F., Jaime L. (2017). IRPL: An energy efficient routing protocol for wireless sensor networks. J. Syst. Archit..

[56] Bholowalia P., Kumar A. (2014). EBK-means: A clustering technique based on elbow method and k-means in WSN. Int. J. Comput. Appl..

[57] Yang L., Lu Y., Xiong L., Tao Y., Zhong Y. (2017). A game theoretic approach for balancing energy consumption in clustered wireless sensor networks. Sensors.

[58] Ullah R., Faheem Y., Kim B.S. (2017). Energy and congestion-aware routing metric for smart grid AMI networks in smart city. IEEE Access.

[59] Sankar S., Srinivasan P., Luhach A.K., Somula R., Chilamkurti N. (2020). Energy-aware Grid-based data Aggregation Scheme in Routing Protocol for Agricultural Internet of Things. Sustain. Comput. Inform. Syst..

